# Upregulation of Bone Morphogenetic Protein-2 Synthesis and Consequent Collagen II Expression in Leptin-stimulated Human Chondrocytes

**DOI:** 10.1371/journal.pone.0144252

**Published:** 2015-12-04

**Authors:** Shun-Fu Chang, Rong-Ze Hsieh, Kuo-Chin Huang, Cheng Allen Chang, Fang-Yao Chiu, Hsing-Chun Kuo, Cheng-Nan Chen, Yu-Ping Su

**Affiliations:** 1 Department of Medical Research and Development, Chang Gung Memorial Hospital Chiayi Branch, Chiayi, Taiwan; 2 Department of Orthopaedics, Chang Gung Memorial Hospital Chiayi Branch, Chiayi, Taiwan; 3 Department of Biomedical Imaging and Radiological Sciences, National Yang-Ming University, Taipei, Taiwan; 4 Department of Orthopaedics and Traumatology, Taipei Veterans General Hospital, Taipei, Taiwan; 5 School of Medicine, National Yang-Ming University, Taipei, Taiwan; 6 Department of Nursing, Chang Gung University of Science and Technology, Chiayi, Taiwan; 7 Department of Biochemical Science and Technology, National Chiayi University, Chiayi, Taiwan; Institute for Nutritional Sciences, CHINA

## Abstract

Bone morphogenetic proteins (BMPs) play positive roles in cartilage development, but they can barely be detected in healthy articular cartilage. However, recent evidence has indicated that BMPs could be detected in osteoarthritic and damaged cartilage and their precise roles have not been well defined. Extremely high amounts of leptin have been reported in obese individuals, which can be associated with osteoarthritis (OA) development. The aim of this study was to investigate whether BMPs could be induced in human primary chondrocytes during leptin-stimulated OA development and the underlying mechanism. We found that expression of BMP-2 mRNA, but not BMP-4, BMP-6, or BMP-7 mRNA, could be increased in human primary chondrocytes under leptin stimulation. Moreover, this BMP-2 induction was mediated through transcription factor-signal transducer and activator of transcription (STAT) 3 activation via JAK2-ERK1/2-induced Ser727-phosphorylation. Of note, histone deacetylases (HDACs) 3 and 4 were both involved in modulating leptin-induced BMP-2 mRNA expression through different pathways: HDAC3, but not HDAC4, associated with STAT3 to form a complex. Our results further demonstrated that the role of BMP-2 induction under leptin stimulation is to increase collagen II expression. The findings in this study provide new insights into the regulatory mechanism of BMP-2 induction in leptin-stimulated chondrocytes and suggest that BMP-2 may play a reparative role in regulating leptin-induced OA development.

## Introduction

Bone morphogenetic proteins (BMPs) were originally identified by their unique ability to induce ectopic bone and cartilage formation *in vivo* [1–2]. BMPs bind and signal through their specific receptors and hence phosphorylate Smad1/5 to stimulate the expressions of target genes [2]. In addition to their differentiation activity in chondrogenesis, BMPs have recently started to receive attention for their roles in both the amelioration and worsening of cartilage damage, including that which occurs during osteoarthritis (OA); BMP-2/4 may be a good candidate with great potential in these processes [2–7]. It has been shown that BMP-2/4 can barely be detected in healthy articular cartilage, but it is highly expressed in osteoarthritic cartilage and joints that have suffered mechanical injury by both chondrocytes and synovial cells, possibly resulting in anabolic development in chondrocytes and/or the formation of osteophytes [2–7]. These studies indicate that BMP might play important role in the control of cartilage structure and composition during injury. However, the precise roles and detailed mechanisms of BMP induction in damaged cartilage have not been taken into account.

Obesity is one of the most significant risk factors for OA development [8]. In addition to its biomechanical loading effects, accumulating evidence has indicated that adipocyte-released adipokines play important roles in this clinical issue [9–10]. Leptin is an adipokine with pleiotropic bioactivities. The original identified function of leptin is energy balance control in cells, but recent studies further report its regulatory activity in inflammation and cartilage damage [11–17]. Leptin binds to its receptor to phosphorylate and activate the specific transcription factor-signal transducer and activator of transcription (STAT) through JAK and/or ERK signaling and hence mediate transcription of various types of genes [15]. The expression of leptin receptors in chondrocytes and other cells in the joints, including the synovium, osteophytes, and bone, have been demonstrated over the last decade [16–17]. In clinical studies, it has been indicated that the concentration of leptin in synovial fluid is positively proportional to OA development [16–17]. In animal studies, it has been shown that extreme obesity in leptin-deficient mice does not increase OA incidence [18]. These results all imply an important role for leptin in OA pathogenesis.

Histone deacetylases (HDACs), enzymes that remove the acetyl group on histones to trigger transcriptional repression, are important epigenetic factors that also regulate the activation of non-histone proteins [19–20]. The HDACs comprise four classes: class I (HDAC1-3 and 8), class II (HDAC4-7, 9 and 10), class III (the sirtuins), and class IV (HDAC11). Accumulating evidence implies that HDACs have control over the development of both OA and rheumatoid arthritis [21]. It has been demonstrated that the HDAC inhibitor Trichostatin A (TSA) inhibits the expression of matrix-degrading enzymes in chondrocytes and suppresses synovial inflammation and cartilage destruction in a mouse arthritis model [22–23]. However, it has also been shown that HDAC activity is decreased during chondrocyte dedifferentiation, and HDAC inhibitors can downregulate type II collagen expression [24]. These studies suggest that HDACs play critical roles in modulating chondrocyte phenotype changes and osteoarthritis progression, but the exact effects of HDACs in articular cartilage remain to be established, especially when cells are exposed to leptin stimulation.

In the present study, we investigated whether BMPs are induced in human primary chondrocytes under leptin-stimulated OA development and the underlying mechanism. We found that leptin increases BMP-2 expression through the transcription factor STAT3 via JAK2-ERK1/2-induced STAT3 Ser727-phosphorylation. HDAC3 and 4 are also involved in leptin-induced BMP-2 expression but through different pathways: HDAC3, but not HDAC4, associates with STAT3 to form a complex. Finally, we demonstrated that the role of BMP-2 expression in leptin-stimulated chondrocytes is to increase the expression level of collagen II. Our findings elucidate the detailed mechanism of BMP-2 expression and indicate a possible reparative role for BMP-2 in leptin-stimulated chondrocytes.

## Materials and Methods

### Materials

Rabbit polyclonal antibodies (pAbs) against Smad1/5 and goat pAb against COX2 were purchased from Santa Cruz Biotechnology (Santa Cruz, CA). Mouse mAbs against HDAC1, HDAC2, and HDAC3, and rabbit pAbs against HDAC4, HDAC6, HDAC7, phospho-Ser727-STAT3, phospho-Tyr705-STAT3, acetyl-STAT3, phospho-STAT5, STAT3, STAT5, ERK, phospho-ERK1/2, phospho-Smad1/5, phospho-JAK1, phospho-JAK2, JAK1, and JAK2 were purchased from Cell Signaling Technology (Beverly, MA). Mouse mAbs against Collagen II were purchased from Chemicon (Temecula, CA). The control siRNA and specific siRNAs of BMP-2, Smad1, Smad5, STAT3, STAT5, JAK1, JAK2, ERK1, ERK2, HDAC3, and HDAC4 were purchased from Invitrogen (Carlsbad, CA). Recombinant human leptin was purchased from PeproTech (Rocky Hill, NJ, USA) and Noggin was purchased from R & D Systems (Minneapolis, MN). BMP-2 ELISA KIT were obtained from “American Diagnostica” (Greenwich, CT, USA). HDAC inhibitor-TSA [21–23] were purchased from Sigma (St Louis, MO). All other chemicals of reagent grade were obtained from Sigma.

### Cell culture

Human primary OA chondrocytes were isolated from OA patients who underwent knee replacement surgery (n = 70; ages 65–82 years). The diagnosis in all patients were based on the American College of Rheumatology criteria for knee OA [25]. The Ethics Committees of Taipei Veterans General Hospital approved the study protocol, and written informed consent was obtained from all patient subjects. Primary chondrocytes were obtained from the non-lesional areas of OA cartilage. The preparation of first passage chondrocytes from human cartilage was performed according to the published report [26]. In brief, the articular cartilage was washed with phosphate buffered saline (PBS) and removed from the underlying bone and then cut into pieces of around 0.5 cm^2^. After sequential enzyme digestion with Pronase (2 mg/mL) (Calbiochem, La Jolla, CA) and then with collagenase I (0.25 mg/mL), the cell suspension was filtered through 100 μm nylon meshes. The isolated chondrocytes were washed repeatedly with PBS and then cultured in DMEM medium containing 10% FBS and 1% antibiotics for 5–7 days before use (~70% confluence). To ensure that the cell phenotype was maintained, experiments were performed using only first-passage cultured chondrocytes. In the experiment, cells were seeded in 6-well plates (~70% confluence) in complete DMEM medium and serum starved for 12 h and then treated with leptin for various time intervals.

### Quantitative real-time PCR

The total RNA was isolated and converted to cDNA. The cDNA was amplified through PCR on a ABI StepOnePlus (Applied Biosystems, Foster City, CA) using SYBR Green PCR Master Mix (Applied Biosystems) with the primers of BMP-2 (sense: 5′-CGCAGCTTCCACCATGAAGAA-3′; antisense: 5′-CCTGAAGCTCTGCTGAGG TGATA-3′), BMP-4 (sense: 5′- AGGAGCTTCCACCACGAAGAAC-3′; antisense: 5′- TGGAAGCCCCTTTCCCAATCAG-3′), BMP-6 (sense: 5′- GTGAACCTGGTGGAGTACG ACAA-3′; antisense: 5′- AGGTCAGAGTCTCTGTGCTGATG-3′), BMP-7 (sense: 5′- CAGCCTGCAAGATAGCCATT-3′; antisense: 5′- AATCGGATCTCTTCCTGCTC-3′), and GAPDH (sense: 5′- AGGTGAAGGTCGGAGTCAAC-3′; antisense: 5′- CCATGTAGTTGA GGTCAATGAAGG-3′) genes. The GAPDH gene expression was used as an internal control. The PCR conditions were optimized to obtain a PCR product with a single peak on melting curve analysis.

### Western blot analysis

Cells were collected and lysed with a RIPA buffer containing 1% NP-40, 0.5% sodium deoxycholate, 0.1% SDS, and a protease inhibitor mixture (PMSF, aprotinin, and sodium orthovanadate). The total cell lysate was separated by SDS-PAGE and transferred onto a nitrocellulose membrane. The membrane was then incubated with the designated antibodies. Immunodetection was performed by using the Western-Light chemiluminescent detection system (Applied Biosystems, Foster City, CA).

### siRNA transfection

For siRNA transfection, cells at 70% confluence were transfected with designed siRNA (40 nM), which caused ~50–80% reductions in expressions of the corresponding proteins (S1 Fig), using the Lipofactamine 2000 Transfection Regents (Invitrogen).

### Immunoprecipitation

The cells were scraped and lysed with a RIPA buffer and the same amount of protein from each sample was incubated with a designated antibody. The immune complex was then incubated with protein A/G agarose and collected by centrifugation. This agarose-bound immunoprecipitates were washed and incubated with boiling sample buffer and then were subjected to SDS-PAGE and Western blotting.

### Chromatin immunoprecipitation (ChIP) assay

ChIP assays were performed using EZ ChIP Assay Kit (Upstate Biologicals) in accordance with recommended protocols. In brief, after stimulation with leptin for indicated time, the cells were fixed with 1% formaldehyde. Immunoprecipitation analysis was carried out using anti-STAT3 antibody. Real-time PCR was performed with primers (sense: 5′-CACTCAATTTCCAGCCTGCT-3′; antisense: 5′-AAACACCTCAATTACAGCG-3′) that amplified the part of the human BMP-2 promoter that contains a putative STAT3 binding site. Then 10% of the chromatin DNA used for immunoprecipitation was similarly subjected to real-time PCR analysis and indicated as an internal control.

### Statistical analysis

Results are expressed as mean ± standard error of the mean (SEM). Statistical analysis was performed by using an independent Student t-test for two groups of data and analysis of variance (ANOVA) followed by Scheffe’s test for multiple comparisons. A *P* value less than 0.05 was considered significant. Results were calculated from at least 3 repetitions generated from individual patients.

## Results

### Leptin induces BMP-2 expression in human primary chondrocytes

**Fig 1:**
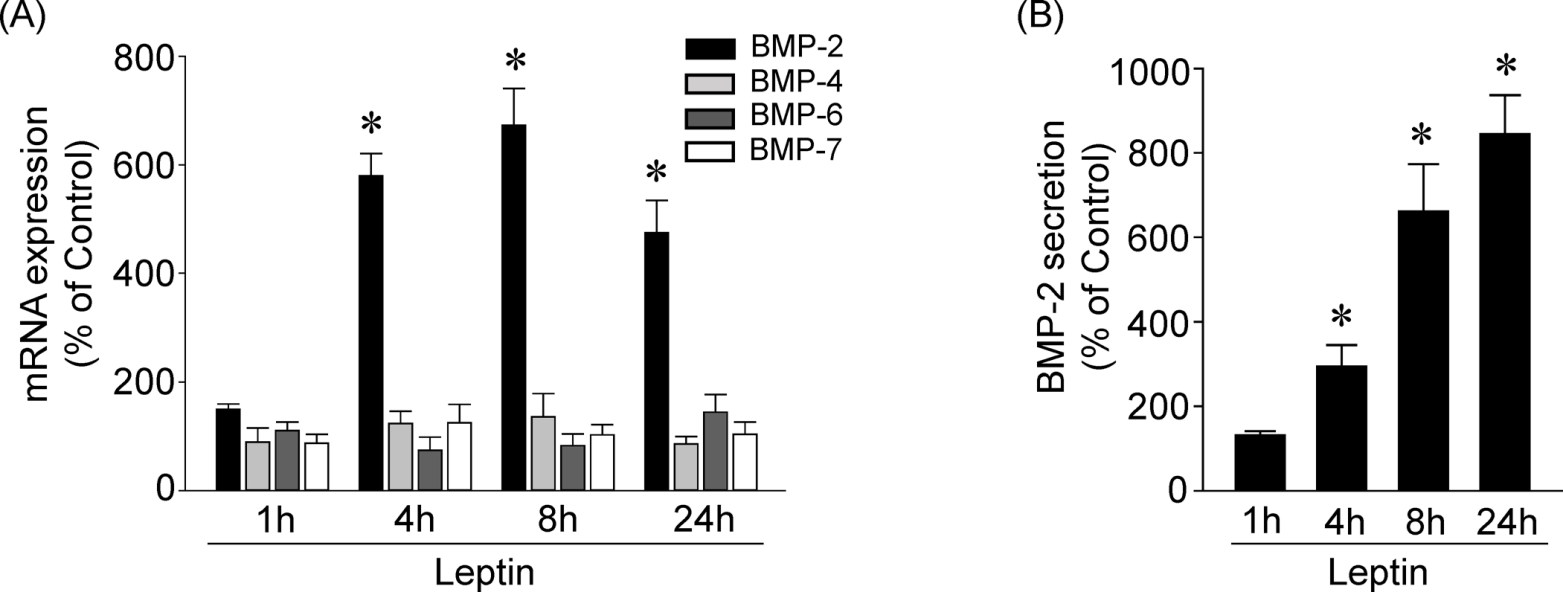
Leptin induces BMP-2 expression in human primary chondrocytes. Cells were kept as controls or stimulated with leptin for 1, 4, 8, and 24 h, and the BMPs mRNA expressions (*A*) and protein secretion (*B*) were determined by real-time PCR and ELISA, respectively. Data in (*A*) and (*B*) are mean ± SEM from three independent experiments. *, *P* < 0.05 *vs*. untreated control cells.

We examined whether leptin induces BMP expression in human primary chondrocytes. Cells were kept as controls or treated with leptin (5 ng/ml) for 1, 4, 8, and 24 h, and their expression of BMP-2, BMP-4, BMP-6 and BMP-7 mRNA was examined. Treatment of cells with leptin resulted in significant increases (within 4 h) in BMP-2 mRNA expression, which reached a maximal level of approximately 6.5 times that of untreated controls within 8 h and then slightly declined but remained elevated after 24 h of treatment (Fig 1A). The expression of BMP-4, BMP-6 and BMP-7 mRNA did not increase in leptin-stimulated cells. Moreover, leptin also induced BMP-2 protein secretion into the medium within 4 h, which persisted for 24 h, compared with untreated control cells (Fig 1B).

### Leptin-induced BMP-2 mRNA expression is mediated by JAK2-ERK1/2 signaling in human primary chondrocytes

**Fig 2:**
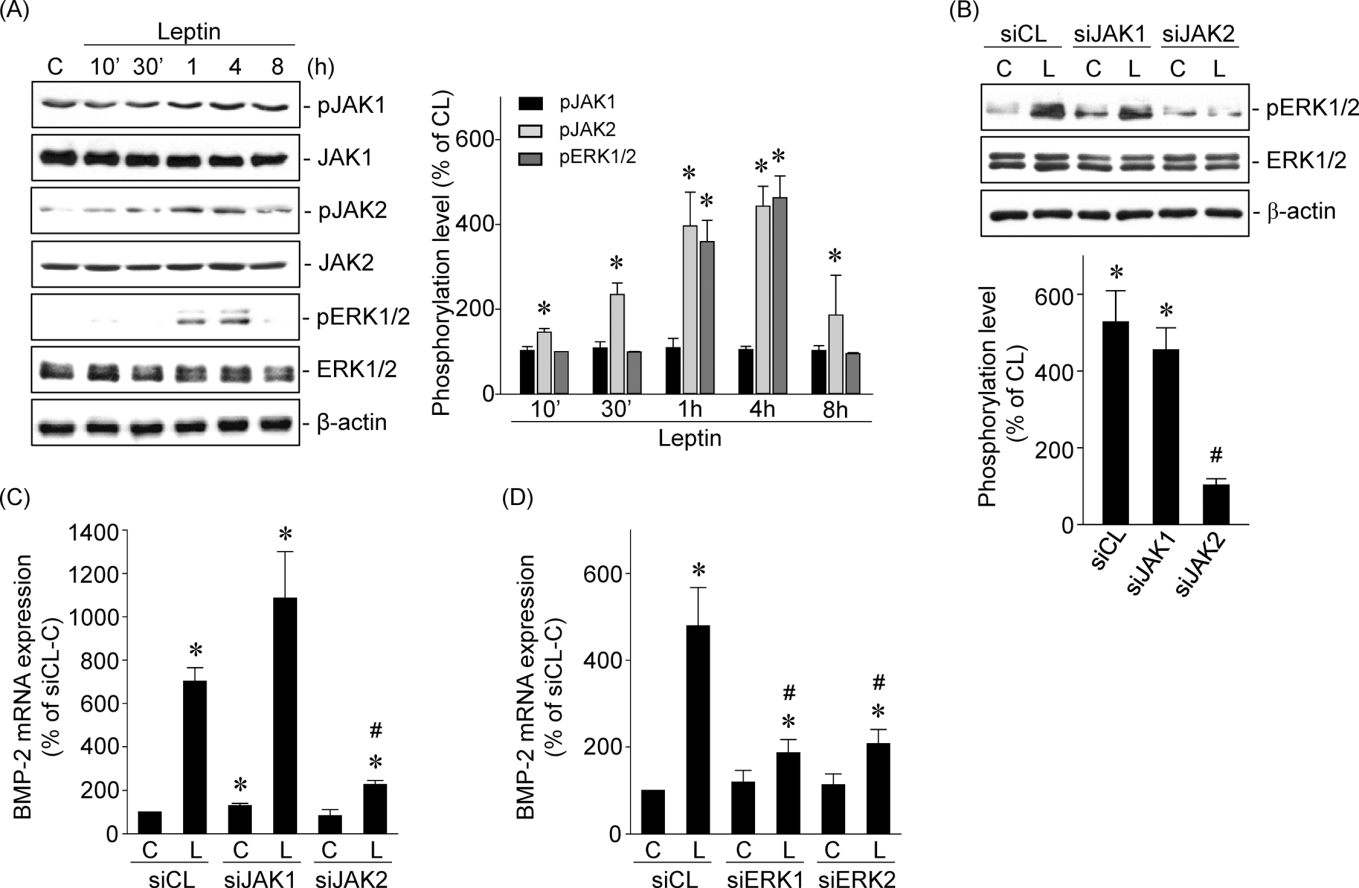
Leptin-induced BMP-2 mRNA expression is mediated by JAK2-ERK1/2 signaling in human primary chondrocytes. (*A*) Cells were kept as controls or treated with leptin for 10’, 30’, 1, 4, and 8 h, and the phosphorylations of JAK1/2 and ERK1/2 were determined by Western blot. (*B*-*D*) Cells were transfected with (*B*-*C*) JAK1- or JAK2-specific siRNA or (*D*) ERK1- or ERK2-specific siRNA and then kept as controls or treated with leptin for 4 h. (*B*) The phosphorylation of ERK1/2 was determined by Western blot and (*C*-*D*) the BMP-2 mRNA expression was determined by real-time PCR. Results in (*A*) and (*B*) are representative of three independent experiments with similar results. Data in (*C*) and (*D*) are mean ± SEM from three independent experiments. *, *P* < 0.05 *vs*. siCL/control or untreated cells. #, *P* < 0.05 *vs*. siCL/leptin-treated cells.

The JAK and ERK pathways are known as leptin-directed downstream signaling pathways [15]. We investigated the roles of JAK1/2 and ERK1/2 in leptin-induced BMP-2 mRNA expression in human primary chondrocytes. Cells stimulated with leptin (5 ng/ml) induced a rapid and transient increase in JAK2 (not JAK1) and ERK1/2 phosphorylation within 1 h compared with untreated control cells (Fig 2A). The increased levels of JAK2 and ERK1/2 phosphorylation declined after 8 h of stimulation. Transfection of cells with JAK2-specific (not JAK1-specific) siRNA significantly inhibited the leptin-induced ERK1/2 phosphorylation (Fig 2B) and BMP-2 mRNA expression (Fig 2C). Transfection of cells with ERK1- or ERK2-specific siRNA also significantly inhibited the leptin-induced BMP-2 mRNA expression (Fig 2D).

### STAT3 regulates leptin-induced BMP-2 mRNA in human primary chondrocytes

**Fig 3:**
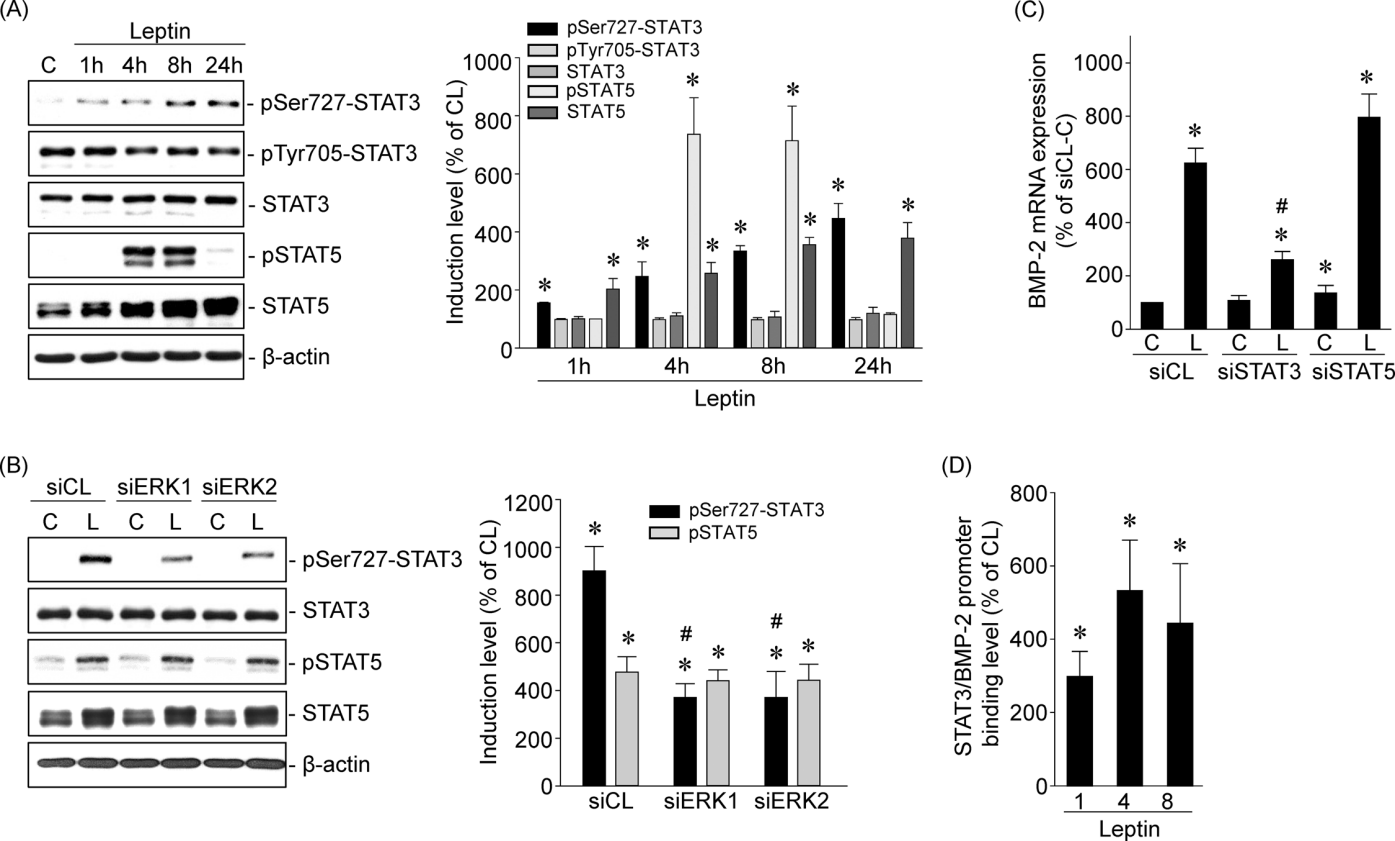
STAT3 regulates leptin-induced BMP-2 mRNA in human primary chondrocytes. (*A*) Cells were kept as controls or treated with leptin for 1, 4, 8, and 24 h, and the phosphorylations of STAT3 and STAT5 were determined by Western blot. (*B-C*) Cells were transfected with (*B*) ERK1- or ERK2-specific siRNA or (*C*) STAT3- or STAT5-specific siRNA and then kept as controls or treated with leptin for 4 h. (*B*) The phosphorylations of STAT3 and STAT5 were determined by Western blot and (*C*) the BMP-2 mRNA expression was determined by real-time PCR. (*D*) Cells were kept as controls or treated with leptin for 1, 4, and 8 h, and the STAT3-BMP-2 promoter binding activity was analyzed by chromatin immunoprecipitation and real-time PCR. Results in (*A*) and (*B*) are representative of three independent experiments with similar results. Data in (*C*) and (*D*) are mean ± SEM from three to four independent experiments. *, *P* < 0.05 *vs*. siCL/control or untreated cells. #, *P* < 0.05 *vs*. siCL/leptin-treated cells.

STATs play a critical role in mediating leptin-regulated gene transcription [15]. Treatment of human primary chondrocytes with leptin (5 ng/ml) induced increases in STAT3 phosphorylation at Ser727, but not Tyr705, within 1 h that persisted until 24 h. Leptin induced STAT5 phosphorylation at Tyr694 within 4 h, which then persisted until 8 h and declined thereafter. Moreover, leptin also induced STAT5 protein expression within 4 h that persisted until 24 h (Fig 3A). Transfection of cells with ERK1- or ERK2-specific siRNA significantly inhibited leptin-induced STAT3 Ser727-phosphorylation, but not that of Tyr705 (Fig 3B). Moreover, transfecting cells with STAT3- or STAT5-specific siRNA showed that only STAT3-specific siRNA transfection abolished leptin-induced BMP-2 mRNA expression (Fig 3C). Chromatin immunoprecipitation (ChIP) assays using an antibody against STAT3 and primers against the putative STAT3-binding element of the BMP-2 promoter demonstrated that exposure of cells to leptin (5 ng/ml) for 1, 4, or 8 h induced *in vivo* STAT3 binding to the STAT3-binding element of the BMP-2 promoter in human primary chondrocytes (Fig 3D).

### HDAC3/4 mediate leptin-induced BMP-2 mRNA expression in human primary chondrocytes

**Fig 4:**
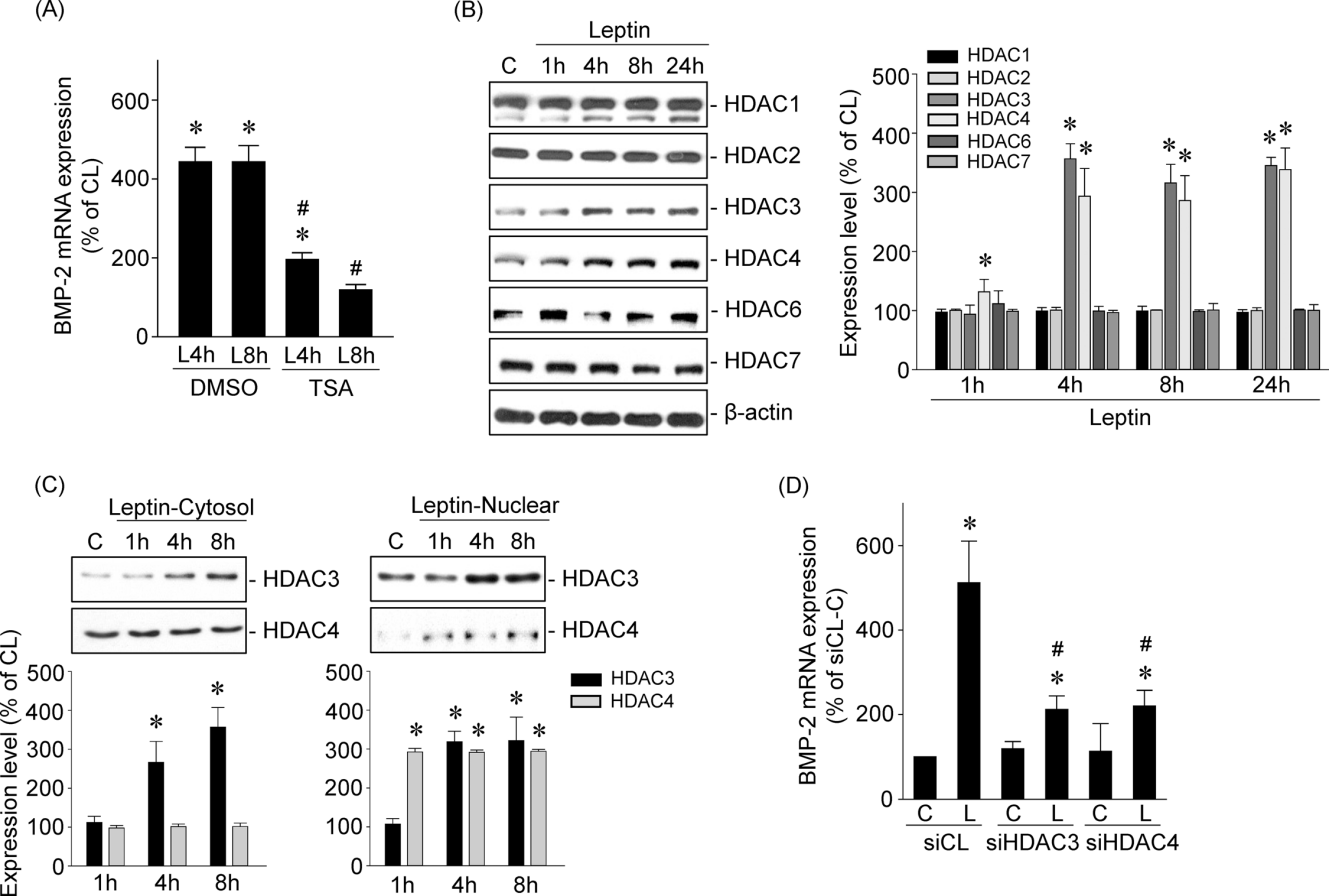
HDAC3/4 mediates leptin-induced BMP-2 mRNA expressions in human primary chondrocytes. (*A*) Cells were pretreated with DMSO or TSA and then kept as controls or treated with leptin for 4 and 8 h. The BMP-2 mRNA expression was determined by real-time PCR. (*B–C*). Cells were kept as controls or treated with leptin for 1, 4, 8 and 24 h. (*B*) The HDACs expressions were determined by Western blot. (*C*) The subcellular localization of HDAC3 and 4 were determined by cell fractionation assay and Western blot. (*D*) Cells were transfected with HDAC3- or HDAC4-specific siRNA and then kept as controls or treated with leptin for 4 h. The BMP-2 mRNA expression was determined by real-time PCR. Data in (*A*) and (*D*) are mean ± SEM from three independent experiments. *, *P* < 0.05 *vs*. DMSO/control, siCL/control or untreated cells. #, *P* < 0.05 *vs*. DMSO/ or siCL/leptin-treated cells. Results in (*B*) and (*C*) are representative of three independent experiments with similar results.

Pretreating cells with DMSO or TSA (1 μM), an HDAC activity inhibitor, and then keeping them as controls or stimulating them with leptin (5 ng/ml) for 4 or 8 h, showed that HDAC activity inhibition blocked leptin-induced BMP-2 mRNA expression in human primary chondrocytes (Fig 4A). Next, we determined which HDAC members were involved in mediating leptin’s effect on BMP-2 mRNA expression. Human primary chondrocytes were kept as controls or treated with leptin (5 ng/ml) for 1, 4, 8, or 24 h, and the expression of class I (HDAC1/2/3) and class II (HDAC4/6/7) HDACs was examined. Treatment of cells with leptin induced HDAC3 and 4 expression within 4 h and that persisted until 24 h compared with untreated control cells (Fig 4B). Cellular fractionation assays, involving separation of cytoplasmic and nuclear fractions of cell lysates derived from leptin-treated cells, demonstrated that leptin induced sustained increases in HDAC3 expression in both the cytoplasm and nucleus and HDAC4 expression in the nucleus (Fig 4C). Moreover, transfecting cells with HDAC3- or 4-specific siRNA also inhibited leptin-induced BMP-2 mRNA expression (Fig 4D) in human primary chondrocytes.

### STAT3-HDAC3 and HDAC4 are divergent pathways mediating leptin-induced BMP-2 mRNA expression in human primary chondrocytes

**Fig 5:**
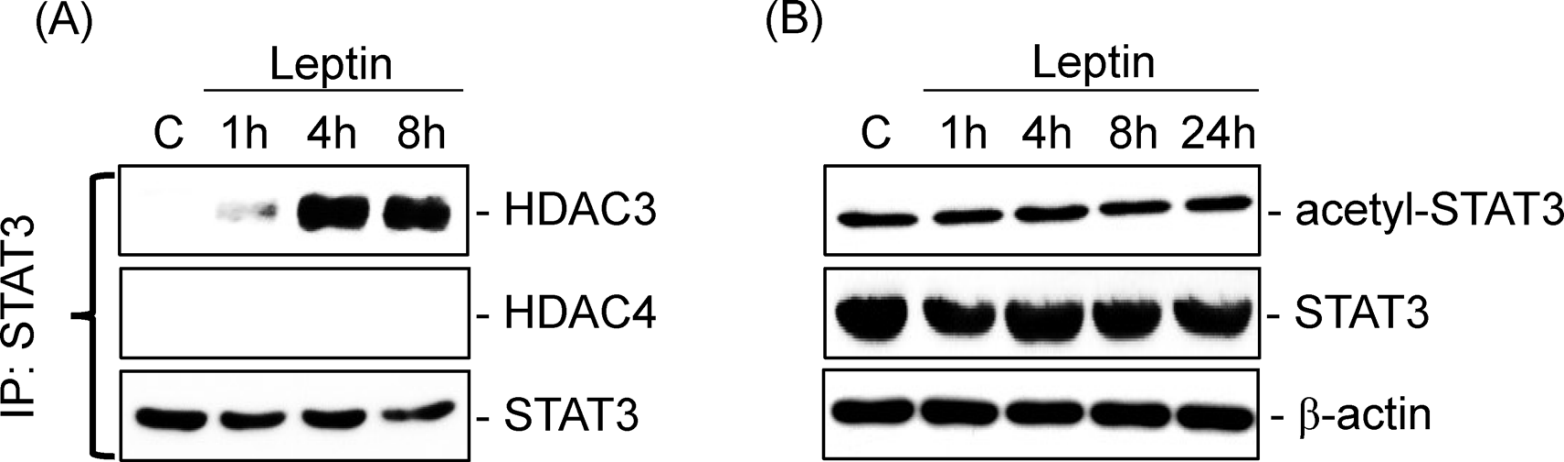
STAT3-HDAC3 and HDAC4 are divergent pathways to mediate leptin-induced BMP-2 mRNA expression in human primary chondrocytes. Cells were kept as controls or treated with leptin for 1, 4, 8, and 24 h. (*A*) The associations of STAT3 with HDAC3 and HDAC4 were determined by an immunoprecipitation assay and Western blot and (*B*) the STAT3 acetylation was determined by Western blot. Results are representative of three independent experiments with similar results.

Next, we determined whether HDAC3/4 and STAT3 cooperatively regulate BMP-2 mRNA expression under leptin stimulation. Human primary chondrocytes were kept as controls or treated with leptin (5 ng/ml) for 1, 4, or 8 h. Cell extracts were immunoprecipitated with an antibody against STAT3 followed by Western blot analysis with an antibody against HDAC3 or 4. Leptin induced rapid increases in the association of HDAC3 with STAT3 within 1 h, as shown by their coimmunoprecipitation, in comparison with untreated control cells (Fig 5A). In contrast, leptin did not induce an association between HDAC4 and STAT3 in human primary chondrocytes. Further, a STAT3 deacetylation assay using Western blotting showed that deacetylation of STAT3 was not increased by treating cells with leptin for 1, 4, 8, or 24 h, even under the formation of the HDAC3 and STAT3 complex (Fig 5B).

### BMP-2 induction in leptin-stimulated human primary chondrocytes increases collagen II expression

**Fig 6:**
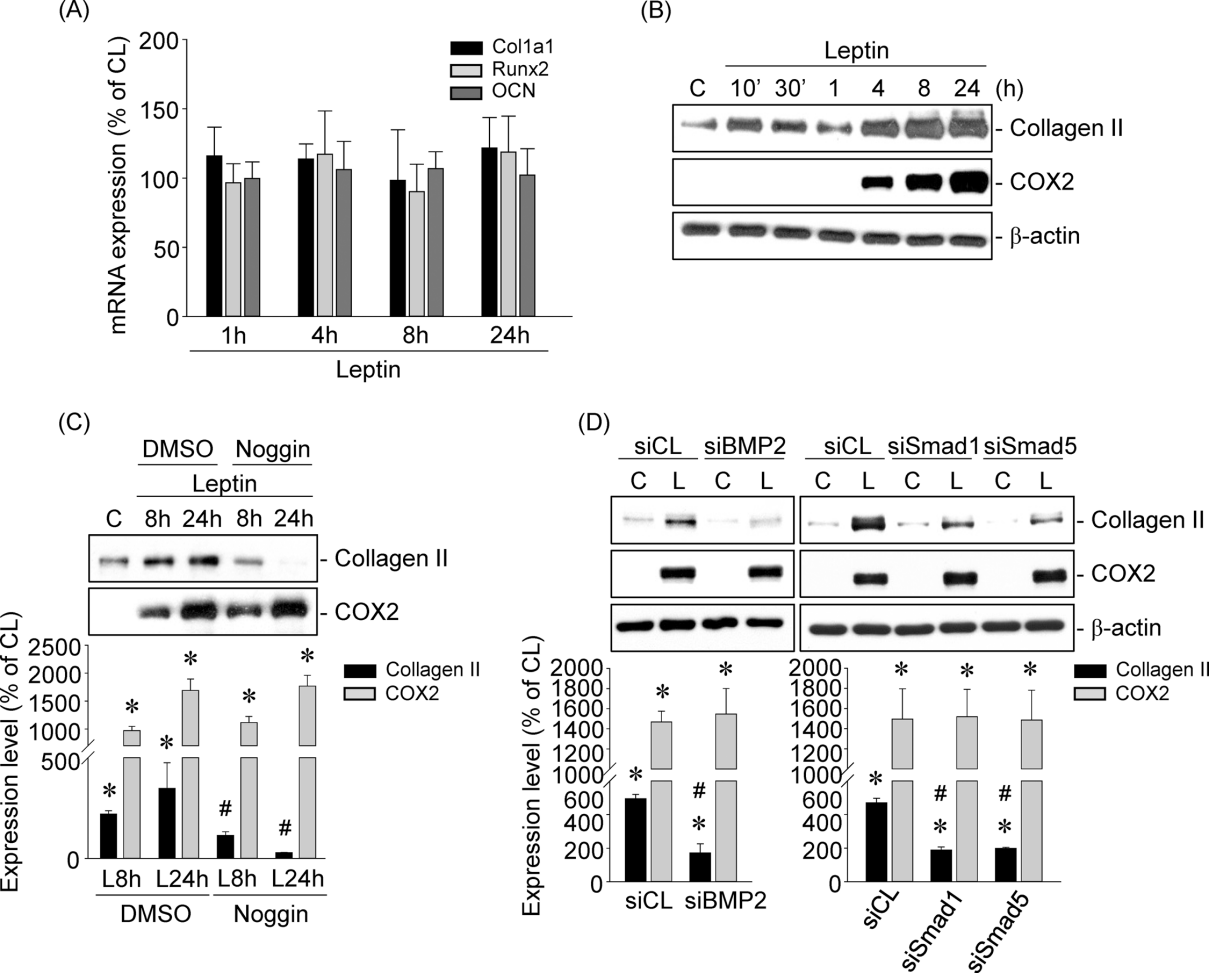
BMP-2 induction in leptin-stimulated human primary chondrocytes increases collagen II expression. (*A-B*) Cells were kept as controls or stimulated with leptin for 10’, 30’, 1, 4, 8, and 24 h. (*C-D*) Cells were (*C*) pretreated with Noggin or (*D*) transfected with BMP-2-, Smad1-, or Smad5-specific siRNA and then kept as controls or treated with leptin for 8 and 24 h. (*A*) The mRNA expressions of col1a1, runx2, and osteocalcin were determined by real-time PCR. (*B*-D) The protein expressions of collagen II and COX2 were determined by Western blot. Data in (*A*) are mean ± SEM from three independent experiments. Results in (*B-D*) are representative of three independent experiments with similar results. *, *P* < 0.05 *vs*. untreated cells. #, *P* < 0.05 *vs*. siCL/leptin-treated cells.

Finally, we determined whether the role of leptin-induced BMP-2 expression in human primary chondrocytes is to drive the cells into the osteoblast lineage and hence promote osteophyte formation or to alter the metabolic homeostasis of chondrocytes. Cells were kept as controls or treated with leptin (5 ng/ml) for 10’, 30’, 1, 4, 8, or 24 h, and the expression of col1a1, runx2 and osteocalcin (osteoblast markers) mRNA and collagen II (anabolic marker) and COX2 (catabolic marker) protein were examined. Stimulation of cells with leptin did not affect the mRNA expression of col1a1, runx2 or osteocalcin (Fig 6A). However, cells stimulated with leptin simultaneously induced both collagen II and COX2 protein expression after 4 h that persisted until 24 h compared with untreated control cells (Fig 6B). Treating cells with Noggin (100 ng/mL), a specific antagonist that binds to BMPs to block their binding to BMP receptors, blocked leptin-induced collagen II, but not COX2, expression (Fig 6C). Moreover, transfecting cells with BMP-2-, Smad1-, or Smad5-specific siRNA further showed that the gene knockdown of BMP-2 and Smad1/5 abolished only leptin-induced collagen II expression (Fig 6D).

## Discussion

**Fig 7:**
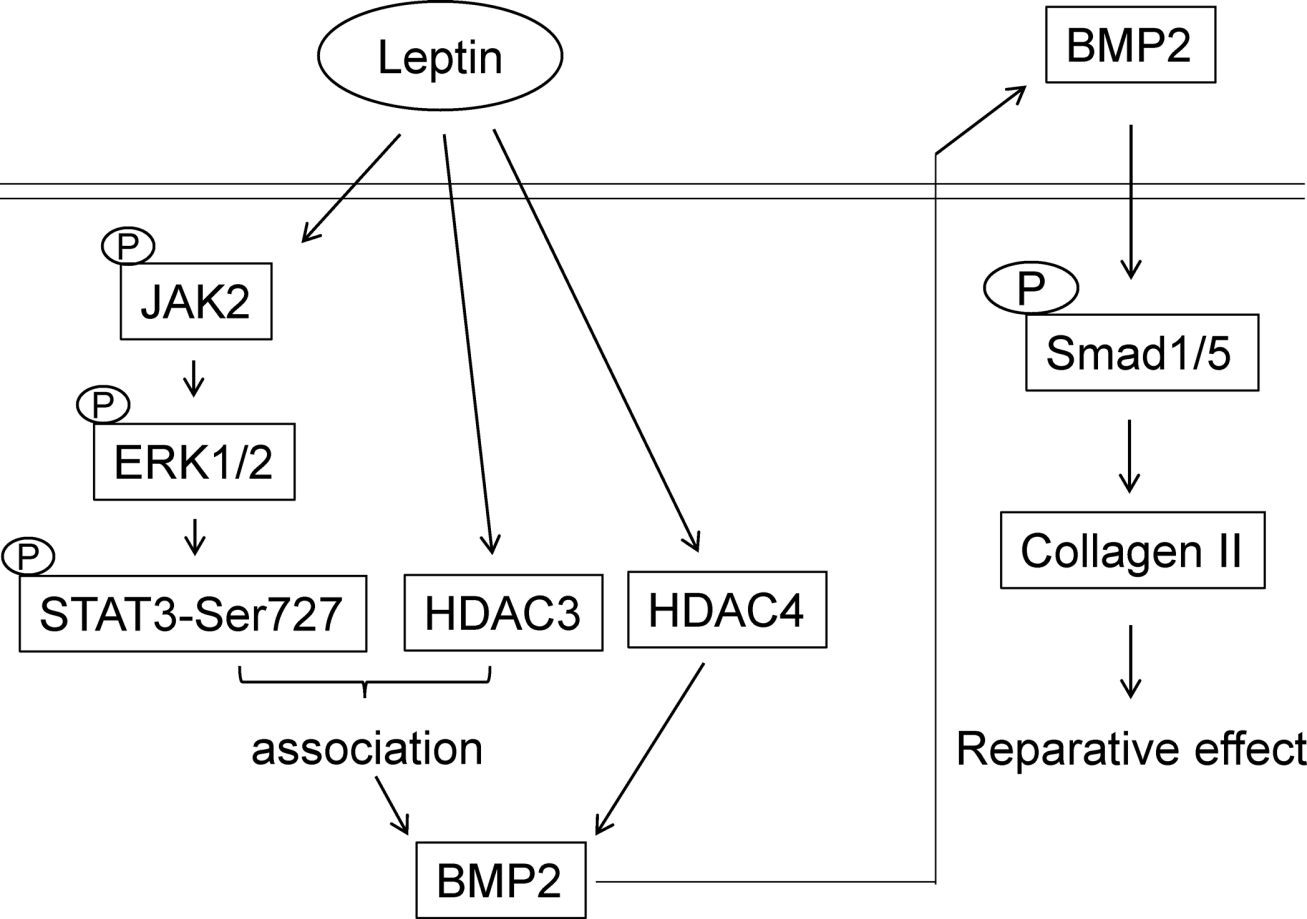
Schematic representation of the signaling pathways regulating leptin-induced BMP-2 and consequent collagen II expressions in human chondrocytes.

This study has elucidated the mechanism whereby leptin upregulates BMP-2 synthesis and consequently increases collagen II expression in human primary chondrocytes (summarized in Fig 7). The systematic experiments demonstrated that (*i*) leptin induces expression of BMP-2, but not that of BMP-4, BMP-6, or BMP-7, in human primary chondrocytes. (*ii*). Leptin-induced BMP-2 expression is regulated by JAK2-ERK1/2-induced STAT3 Ser727-phosphorylation. (*iii*) Both HDAC3 and 4 are involved in regulating leptin-enhanced BMP-2 mRNA expression but through different pathways: HDAC3, but not HDAC4, associates with STAT3 to cooperatively regulate BMP-2 mRNA expression. (i*v*) The role of leptin-enhanced BMP-2 synthesis in human primary chondrocytes is to increase collagen II expression. Our findings provide new insights into the regulatory mechanism and potential reparative effect of BMP-2 synthesis in human chondrocytes under leptin-initiated OA development.

The regulation of various stages of chondrogenesis by BMPs has been well defined [27–29]. However, recently, accumulating evidence has indicated that some BMPs are detected in osteoarthritic adult cartilage. These studies have further implicated that the expression of BMPs in OA lesional areas might play important roles in the control of cartilage structure and composition, for example by improving cartilage integrity and promoting of osteophyte formation [2–7, 30–31]. Our data revealed the upregulation of BMP-2 synthesis in human primary chondrocytes when leptin induced OA marker expression. Further experiments showed that leptin did not affect the mRNA expression of col1a1, runx2, or osteocalcin (osteoblast markers), but it did increase the protein expression of collagen II (an anabolic marker) through BMP-2. Therefore, although we cannot completely rule out a possible role for BMP-2 in promoting osteophyte formation under leptin stimulation, the results from our current study provide evidence that BMP-2 might contribute to the reparative mechanism to neutralize the proinflammatory cytokines’ catabolic effects, including leptin. However, we suggest that BMP-2 activity alone is not sufficient to adequately protect against the destruction of cartilage. This might also be the reason why the rescue response arises only in the early phase of OA under proinflammatory cytokine stimulation and why OA is a deleterious and irreversible disease [17]. Hence, although BMP-2 has been suggested to be a potential rescue factor that eases OA progression by previous studies and by our present study, it remains to be further determined how BMP-2 could be applied clinically in the future.

STATs are the major transcription factors of leptin signaling that regulate target gene transcription [15]. In chondrocytes, both STAT3 and STAT5 have been reported to increase the expression of matrix metalloproteinases under leptin stimulation [32]. In addition, STAT3 has also been demonstrated to regulate the expression of multiple genes in leptin-induced chondrocytes, e.g., type X collagen and Wnt-specific receptors (Frizzled receptors) [33–34]. In the present study, we found that leptin induced both STAT3 and STAT5 phosphorylation and increased STAT5 expression in human primary chondrocytes. However, our further examination elucidated that leptin-induced BMP-2 expression was regulated only by STAT3, and not STAT5. We hence suggest that the role of STAT5 induction in leptin-induced chondrocytes may be in regulating the catabolic effects of leptin in these cells. Our results showed that STAT3 was activated in leptin-induced BMP-2 expression by JAK2-ERK1/2-induced STAT3 Ser727-phosphorylation, but not Tyr705-phosphorylation. The phosphorylation of residues Tyr705 and Ser727 on STAT3 through the JAK and ERK pathways has been regarded as characteristic of STAT3 activation, increasing its transcription activity. It is generally thought that STAT3 activation requires dimerization through Tyr705-phosphorylation, and its transcriptional activity can be further modulated by Ser727-phosphorylation in a manner that is dependent on the cell type and the stimulus used [35–36]. However, accumulating evidence has indicated that the functional regulation of Ser727-phopshorylation on STAT3 is more complex than traditionally expected [36–38]. For example, Sakaguchi M et al. elucidated that STAT3 Ser727-phosphorylation is not necessarily a secondary event after Tyr705-phosphorylation in melanoma cells and that this Ser727-only phosphorylation on STAT3 regulates STAT3 nuclear translocation and cell survival [36]. Zhou Z et al. also indicated that the leptin-enhanced MCP-1 mRNA levels are due to the increased phosphorylation of STAT3 on Ser727 but not on Tyr705 in adipose stem cells [39]. These reports could explain our results indicating that leptin-induced STAT3 activation occurs only through Ser727 phosphorylation. Hence, these findings indicate that Ser727-phosphorylation only on STAT3 is one of the important mechanisms of leptin’s effect and also one of the general mechanisms of STAT3 transcription activity induction.

HDACs play a critical role in removing the Lys acetylation of histone and non-histone proteins and consequently regulating their activity [19–20]. Mounting evidence indicates that HDACs activity is involved in the control of chondrocyte gene expression and function [21–24]; however, which HDACs modulate these processes and their precise role in modulating chondrocyte responses to leptin remain poorly understood. Our present data shows that both HDAC3 and 4 are involved in leptin-induced BMP-2 mRNA expression but through distinct pathways: HDAC3, but not HDAC4, associates with STAT3. These results suggest that HDAC4 might interact with other transcription factors and/or regulate histone proteins to increase BMP-2 mRNA expression, warranting further exploration. Previous studies have shown that STAT3 is a substrate protein of HDAC3 and hence increases STAT3 phosphorylation and activation in multiple cancers [40–42]. We therefore examined the impact of HDAC3 activity on STAT3 acetylation. However, inconsistently with previous studies in cancer cells, we did not observe the downregulation of STAT3 acetylation after STAT3-HDAC3 complex formation in human primary chondrocytes in response to leptin. We propose the possibility that their interaction might serve to bring HDAC3 to the promoter of the BMP-2 gene, where it could modify chromatin. Recently, some evidence has also indicated that HDAC inhibitor suppression of JAK2/STAT3 signaling in cancer cells occurs through inducing the acetylation of histones connected to the promoter of SOCS3, a potent feedback inhibitor of JAK2/STAT signaling [43–44]. Thus, the question of whether HDAC4 and the HDAC3/STAT3 complex regulate the deacetylation of other transcriptional cofactors and/or chromatin factors and consequently mediate leptin-induced BMP-2 expression in human chondrocytes will require ongoing study.

The present study has reported that leptin increases BMP-2 expression to initiate a potent reparative effect when triggering catabolic damage in human chondrocytes. These results provide new insights into the understanding of the complexities of leptin-initiated OA development. Accumulating studies indicate that leptin and its associated signaling may serve as promising novel drug targets and appear to be potential strategies in the therapy of OA, especially in obese patients [17, 45]. Our data concerning the anabolic activity of leptin may contribute new ideas to drug design and development for treating OA progression. Moreover, our findings also suggest the possible clinical application of BMP-2 and should be taken into account in OA therapy in the future.

## Supporting Information

S1 Fig The blocking effect of specific siRNAs on the respective protein expression in human primary chondrocytes. Cells were transfected with siCL or designed siRNAs for 48 h and their respective protein expression were determined by Western blot. Results are representative of three independent experiments with similar results.
(TIFF)
