# Supplementary figures and images for: Upregulation of Bone Morphogenetic Protein-2 Synthesis and Consequent Collagen II Expression in Leptin-stimulated Human Chondrocytes
Source: PLoS One. 2015 Dec 4;10(12):e0144252. doi: 10.1371/journal.pone.0144252 (PMC4670096; doi:10.1371/journal.pone.0144252)

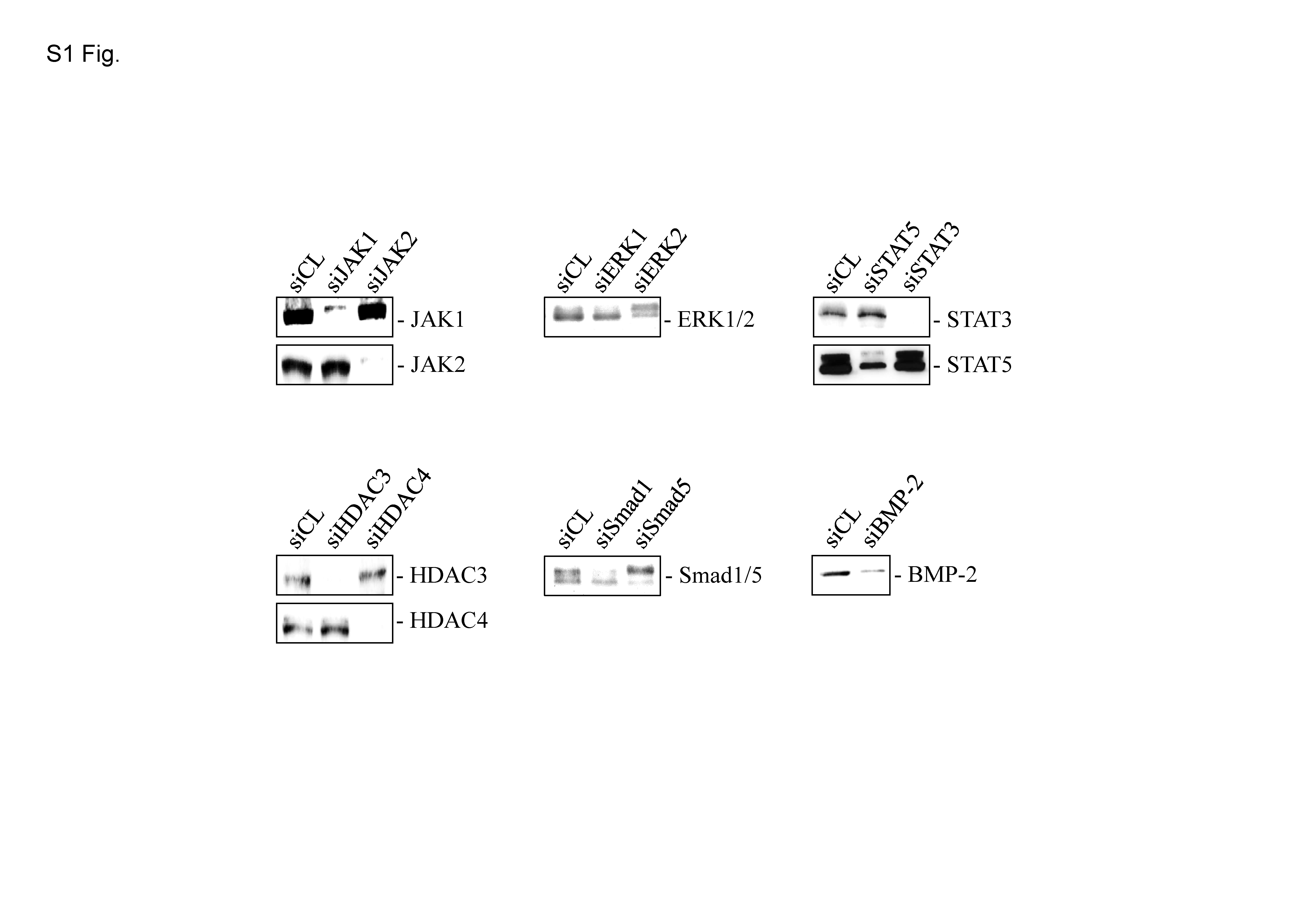

Supplement: S1 Fig — Cells were transfected with siCL or designed siRNAs for 48 h and their respective protein expression were determined by Western blot. Results are representative of three independent experiments with similar results. (TIFF) [file pone.0144252.s001.tiff]
